# *KRAS* Promoter G-Quadruplexes from Sequences of Different Length: A Physicochemical Study

**DOI:** 10.3390/ijms22010448

**Published:** 2021-01-05

**Authors:** Federica D’Aria, Bruno Pagano, Luigi Petraccone, Concetta Giancola

**Affiliations:** 1 Department of Pharmacy, University of Naples Federico II, Via D. Montesano 49, 80131 Naples, Italy; federica.daria@unina.it (F.D.); bruno.pagano@unina.it (B.P.); 2 Department of Chemical Sciences, University of Naples Federico II, Via Cintia 4, 80126 Naples, Italy; luigi.petraccone@unina.it

**Keywords:** *KRAS* G-quadruplex, CD spectroscopy, differential scanning calorimetry, thermodynamic stability

## Abstract

DNA G-quadruplexes (G4s) form in relevant genomic regions and intervene in several biological processes, including the modulation of oncogenes expression, and are potential anticancer drug targets. The human *KRAS* proto-oncogene promoter region contains guanine-rich sequences able to fold into G4 structures. Here, by using circular dichroism and differential scanning calorimetry as complementary physicochemical methodologies, we compared the thermodynamic stability of the G4s formed by a shorter and a longer version of the *KRAS* promoter sequence, namely 5′-AGGGCGGTGTGGGAATAGGGAA-3′ (*KRAS* 22RT) and 5′-AGGGCGGTGTGGGAAGAGGGAAGAGGGGGAGG-3′ (*KRAS* 32R). Our results show that the unfolding mechanism of *KRAS* 32R is more complex than that of *KRAS* 22RT. The different thermodynamic stability is discussed based on the recently determined NMR structures. The binding properties of TMPyP4 and BRACO-19, two well-known G4-targeting anticancer compounds, to the *KRAS* G4s were also investigated. The present physicochemical study aims to help in choosing the best G4 target for potential anticancer drugs.

## 1. Introduction

The discovery of G-quadruplex (G4) arrangements in G-rich DNA sequences has shed light on possible new biological functions for DNA. G4s are nucleic acid structures formed in relevant genomic regions, such as telomeres and oncogene promoters [1]. G4 structures originate by stacking interactions of G-tetrads, formed by four guanines, which shares Hoogsteen hydrogen bonds [2,3]. A few years ago, cellular studies proved the in vivo existence of G4s, thus corroborating their involvement in biological processes of living cells [4].

A growing body of evidence supports the notion that G4s formed by promoter sequences are involved in the regulation of gene expression [5,6]. Specifically, G4s were found in oncogene promoter regions that are crucial for the development of cancer [7]. For these reasons, targeting G4s in gene promoter regions can result into a very effective anticancer approach whose huge potential is still to be explored [8,9]. Many gene promoter G4s have biophysical and structural properties that make them good targets for drug design, and their structural diversity suggests that a high degree of selectivity might be possible. However, the application of G4-stabilizing ligands for the modulation of oncogenic activity is still in its infancy, and few compounds have been proven to bind to G4 structures in oncogene promoters [10].

Among G4-forming sequences in gene promoter regions, particularly interesting are those formed in promoter regions of the *Kirsten ras* (*KRAS*) gene. The three genes of the ras (rat sarcoma) family are involved in transmitting signals within cells, encoding for proteins designated as HRas, NRas and KRas. In particular, the *KRAS* oncogene is involved in the pathogenesis of different types of cancers, and its promoter contains G-rich elements able to fold in G4 DNA [11,12,13].

**Figure 1:**
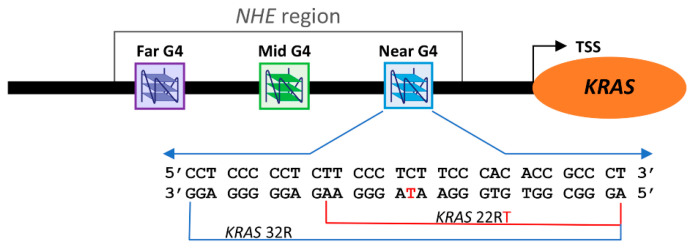
Schematic representation of the Nuclease-Hypersensitive Element (NHE) region of the *KRAS* gene promoter.

Specifically, the promoter of the human *KRAS* gene contains a Nuclease-Hypersensitive Element (NHE), which is crucial for transcription regulation. Xodo and coworkers showed that the NHE contains G-rich sequences able to fold into different G4 structures recognized by several nuclear proteins [14]. Particularly, they studied a 32-nucleotide G4-forming sequence (Figure 1), known as *KRAS* 32R or 32R, and suggested a role for this G4 in transcription. In addition, *KRAS* 32R was found to form almost two conformations, although the structure has not yet been determined at the time. A shorter 22-mer sequence with four G-tracts contained in the 32-mer sequence was also studied as a model for the in silico screening of ligands. The native 22-mer sequence and that with a single G to T (G16→T) mutation, named *KRAS* 22RT, appeared to adopt the same predominant G4 conformation, but the mutated sequence showed better resolved NMR peaks [15]. This G4-forming sequence is also shown in Figure 1.

For *KRAS* 22RT, both an NMR [15] and an X-ray [16] structure were reported. The NMR studies show a parallel monomeric G4 with a thymidine bulge, two single-nucleotide propeller loops and a four-nucleotide loop (Protein Data Bank (PDB): 5I2V). Conversely, the crystal structure shows a parallel 5′-head-to-head asymmetric dimer with extensive poly-A π-stacking interactions observed across the dimer (PDB: 6N65 and 6WCK). Each monomer is quite different from the monomeric structure identified by NMR. The most substantial difference lies in the position of residue T8 that, in the NMR structure, is out and away of the G-tetrad core, whereas in the X-ray structure, forms a 3′-cap over the G-tetrad core. Recently, NMR studies for *KRAS* 32R have been also reported [17]. The authors found that *KRAS* 32R folds into two main parallel conformations in equilibrium with each other. The main difference between them is the presence of a triad at the 3′end that confers to one of the two conformers a major rigidity (PDB: 6SUU and 6T2G).

These two G4-forming sequences are both utilized as a drug target to check their binding interaction with potential new drugs. However, their thermodynamic stability has never been deeply studied with and without drugs beyond the melting temperature stabilization.

With the aim to help in choosing the best *KRAS* G4 target for potential anticancer drugs, herein, we compared the thermodynamic stability of the *KRAS* 32R and *KRAS* 22RT G4s and their interactions with two well-known G4 ligands, TMPyP4 and BRACO-19. Cationic porphyrins are among the most studied molecules for the G4 stabilization [18,19]. The cationic porphyrin TMPyP4 was found to interact with the *KRAS* G4 parallel structure since 2006. Xodo and coworkers showed that the TMPyP4 end-stacks to the *KRAS* 32R by photocleavage and circular dichroism (CD) experiments [20] and may have a role in G4-proteins recognition [21]. Some TMPyP4 derivatives were also found to effectively interact with *KRAS* G4s [22,23]. The other G4 ligand is a tri-substituted acridine molecule, BRACO-19, discovered years ago by Neidle’s group [24]. BRACO-19 was found to bind telomeric G4 with a higher affinity and with a good telomerase inhibitory activity [25]. Its moiety interacts with guanine tetrads by π-π-stacking interactions [26,27]. Recently, BRACO-19 was recently investigated for its interaction with *KRAS* 22RT by CD and Raman spectroscopies [28].

The stability of the two G4s, with and without ligands, was studied by circular dichroism (CD) and differential scanning calorimetry (DSC) and the results discussed also in light of their structures in solution.

## 2. Results

The thermodynamic stability of *KRAS* 32R and *KRAS* 22RT G4s was studied by CD spectroscopy and DSC. The two methodologies work on different concentration scales: DSC measurements need a concentration about two orders of magnitude higher than that of CD measurements. The comparison of the melting temperature (*T*_m_) and enthalpy change values obtained by DSC with those obtained by CD should give information on the molecularity of the unfolding process. Calorimetric unfolding enthalpy, Δ_exp_*H*°, is model-independent, being obtained by the area of the DSC thermogram, whereas the unfolding enthalpy obtained by CD measurements is model-dependent, being calculated by the van ’t Hoff equation, based on the hypothesis of a two-states unfolding process [29]. The van ’t Hoff enthalpy (Δ_v.H__._*H*°) can be also calculated from DSC data [30]. A Δ_exp_*H*°/Δ_v.H._*H*° ratio close to one indicates that the unfolding process involves only two states, the folded and the unfolded G4. On the other hand, a ratio greater than one indicates a more complex unfolding mechanism (which likely includes the formation of unfolding intermediates), which requires a different interpretative model.

In addition, CD measurements are very useful to quickly assess the stabilizing effect of a ligand interacting with a G4 structure. Herein, the potentiality of these two orthogonal techniques was utilized to gain information on the different thermodynamic stability of these G4s and their interactions with two different G4-targeting ligands.

### 2.1. CD Melting Experiments of KRAS 22RT and KRAS 32R

**Figure 2:**
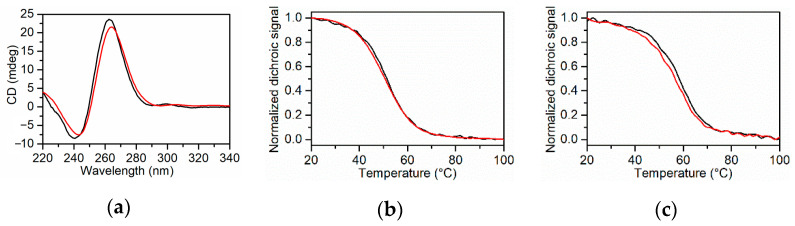
(**a**) Circular dichroism (CD) spectra of *KRAS* 22RT (black line) and *KRAS* 32R (red line). CD melting (black line) and annealing (red line) profiles of (**b**) *KRAS* 22RT and (**c**) *KRAS* 32R at 0.5 °C min^−1^. The buffer solution was 20 mM KH_2_PO_4_/K_2_HPO_4_ with 60 mM KCl and 0.1 mM EDTA at pH 7.0.

CD spectra of *KRAS* 32R and *KRAS* 22R show the characteristic profile of parallel G4 structures (Figure 2a), with a maximum at 260 nm and a minimum at 240 nm [31,32,33]. The CD melting and annealing curves for *KRAS* 22RT and *KRAS* 32R G4s at the scan rate of 0.5 °C min^−1^ are shown in Figure 2b,c, respectively. The melting and annealing curves are superimposable. Moreover, similar curves were obtained at the scan rate of 1 °C min^−1^ (Figure S1), indicating that, in these solution conditions, the folding-unfolding process for the two G4-forming sequences is not kinetically controlled.

The CD melting curves were fitted with a two-states model by the van ’t Hoff analysis, and the melting temperatures along with the enthalpy changes obtained are collected in Table 1. The *T*_m_ values found for *KRAS* 32R and *KRAS* 22RT are in perfect agreement with those previously reported by Salgado et al. (59.9 and 51.8 °C, respectively) in similar solution conditions (20 mM KH_2_PO_4_/K_2_HPO_4_, 60 mM KCl and 0.1 mM EDTA) [15,17]. Three-dimensional melting curves for G4s were also obtained by recording whole CD spectra as a function of temperature (Figure S2). The melting temperatures derived from the analysis of 3D melting curves, accumulated every two degrees, are in perfect agreement with those obtained at a single wavelength at 0.5 or 1 °C min^−1^ heating rates. The *T*_m_ of *KRAS* 32R is greater than that of *KRAS* 22RT, indicating a higher thermal stability of the overall structure.

From this analysis, only a rough comparison between the two enthalpy changes is possible, because these values are model-dependent. A more accurate Δ*H°* determination comes from DSC measurements.

### 2.2. DSC Measurements of KRAS 22RT and KRAS 32R

**Figure 3:**
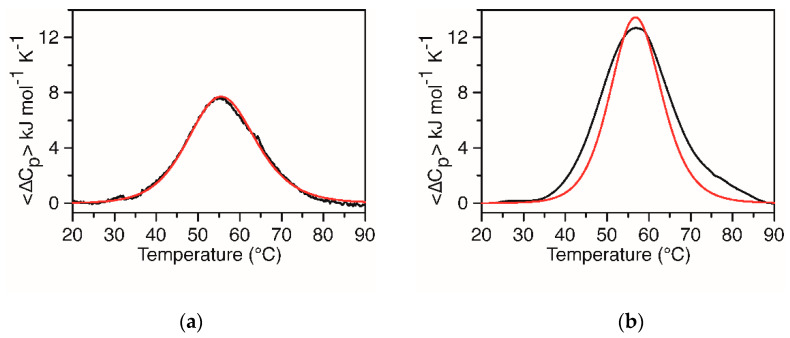
Experimental differential scanning calorimetry (DSC) profiles (black line) and van ’t Hoff calculated curves based on the two-states model (red line) for (**a**) *KRAS* 22RT and (**b**) *KRAS* 32R. The buffer solution was 20 mM KH_2_PO_4_/K_2_HPO_4_ with 60 mM KCl and 0.1 mM EDTA at pH 7.0.

DSC measurements on both the G4-forming sequences were performed to obtain the model-independent thermodynamic analysis [29]. Indeed, Δ_exp_*H*° is directly obtained by integrating the experimental curve of the heat capacity, <Δ*C_p_*>, versus temperature and Δ*S*° by integrating the curve of <Δ*C_p_*>/T versus T. The Gibbs energy change, Δ*G*°, was calculated at 37 °C, assuming a negligible heat capacity change between the native and denatured states. In addition, the van ’t Hoff enthalpy was also calculated from the DSC curves, as previously reported [29]. Figure 3 shows the experimental DSC profiles together with the calculated curves based on the two-states model. Noteworthy, the *KRAS* 22RT curve is well-described by a two-states melting process (Figure 3a), whereas this model is clearly inadequate for the DSC profile of *KRAS* 32R (Figure 3b).

Although the curve of *KRAS* 32R is symmetrical, the maximum of the peak is not sharp and spans between 58 and 59 °C, and the enthalpy change calculated by the two-states model (Δ_v.H._*H*° = 220 kJ mol^−1^) is lower than the experimental calorimetric enthalpy change (Δ_exp_*H*° = 270 kJ mol^−1^).

Therefore, based on the recent NMR studies demonstrating that *KRAS* 32R adopts two major conformations in equilibrium with each other [17], a thermodynamic model was developed for a best fitting of the DSC profile (see the Materials and Methods Section for details), considering the following equilibria:

Q1 ⇆ U

Q2 ⇆ U

where Q1 and Q2 are the two folded G4s, and U is the common unfolded DNA conformation.

**Figure 4:**
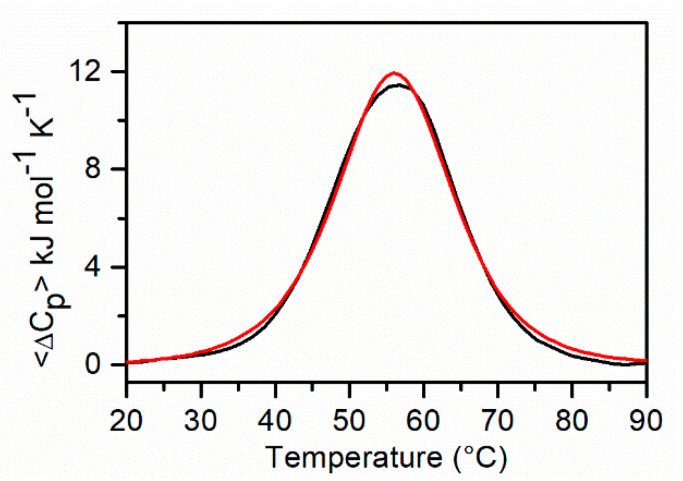
DSC profile of *KRAS* 32R (black line) and the best fitting curve (red line) obtained by the thermodynamic model described in Section 4.4.1.

This model provided a much better fit of the experimental curves (Figure 4), and the corresponding thermodynamic parameters are collected in Table 2. Figure S3 shows the corresponding calculated molar fraction of each species. Interestingly, we found that the total enthalpy measured is close to the fitted value of the Q1 unfolding enthalpy change, as expected if one considers that at low temperatures is present mainly Q1 (99% at 20 °C) and only U at high temperatures. This observation further supports the consistency of the model-derived enthalpies with the experimental value.

Analyzing in detail, the melting temperatures of the two conformers (56.0 and 57.0 °C) are very close to each other, whereas the enthalpy and entropy changes are quite different. The conformer with the higher Δ*H*° and Δ*S*° is stabilized by a greater number of interactions and characterized by a more compact structure. This could be explained looking at the structures identified in the NMR study, which describes one of the conformers capped with a triad (G28, A30 and G31) at the 3′-end. The presence of this triad, together with other peculiar topological arrangements, confers to this conformer an increased thermodynamic stability [17]. Accordingly, we found that this conformer is the most populated (89%) at 37 °C.

### 2.3. Interaction of KRAS 22RT and KRAS 32R with TMPyP4 and BRACO-19

**Figure 5:**
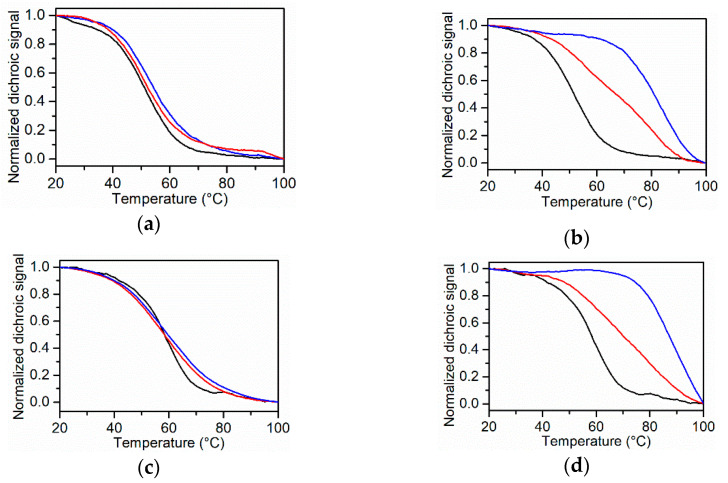
CD melting curves of (**a**) *KRAS* 22RT and (**c**) *KRAS* 32R in the absence (black line) and in the presence of 1 (red line) and 2 equivalents of TMPyP4 (blue line). CD melting curves of (**b**) *KRAS* 22RT and (**d**) *KRAS* 32R in the absence (black line) and in the presence of 1 (red line) and 2 equivalents (blue line) of BRACO-19. The buffer solution was 20 mM KH_2_PO_4_/K_2_HPO_4_ with 60 mM KCl and 0.1 mM EDTA at pH 7.0.

The different stabilizing effects of TMPyP4 and BRACO-19 were studied by CD melting experiments measuring the changes in the melting temperatures of the G4s induced by ligands (Δ*T*_m_). Thermal unfolding was monitored at the wavelength of the maximum CD intensity. The results are shown in Figure 5. In the presence of one and two equivalents of the ligands, no significant variations in the CD spectra of the G4s was detected, indicating a preservation of the overall DNA structure (Figure S4).

The results of the CD melting experiments show that the addition of one and two equivalents of TMPyP4 have a low effect on the G4s’ stability (Figure 5a,c), with a detectable slight increase in the melting temperature observed for one equivalent (Δ*T*_m_ = 2 °C) and two equivalents (Δ*T*_m_ = 3 °C) only on *KRAS* 22RT (Figure 5a). This result is in agreement with our previous studies, which showed that the presence of eight equivalents of TMPyP4 increases the stability of *KRAS* 22RT 11 °C [23].

On the other hand, BRACO-19 significantly increases the stability of both the G4s, with a more marked effect on *KRAS* 32R. For *KRAS* 22RT, the Δ*T*_m_ is 16 °C (Figure 5b), whereas, for *KRAS* 32R, a Δ*T*_m_ is difficult to be estimated, since the unfolding is not complete even at 100 °C, and the half-point of the thermal transition is not accurately determinable in the temperature range analyzed by CD (Figure 5d). For this reason, we performed CD melting experiments also in the presence of one equivalent of BRACO-19. The obtained CD curves show that BRACO-19 enhances the stability of both G4s and seems to promote biphasic profiles for *KRAS* 22RT with two inflection points (Figure 5b). A possible explanation is that BRACO-19 binds to *KRAS* 22RT with a stoichiometry greater than 1:1 (probably 2:1 [34]); thus, the two inflection points could reveal a distribution of the G4 among the complexed and uncomplexed forms. The CD melting curve in the presence of two equivalents of the ligand not showing a biphasic transition supports this hypothesis.

**Figure 6:**
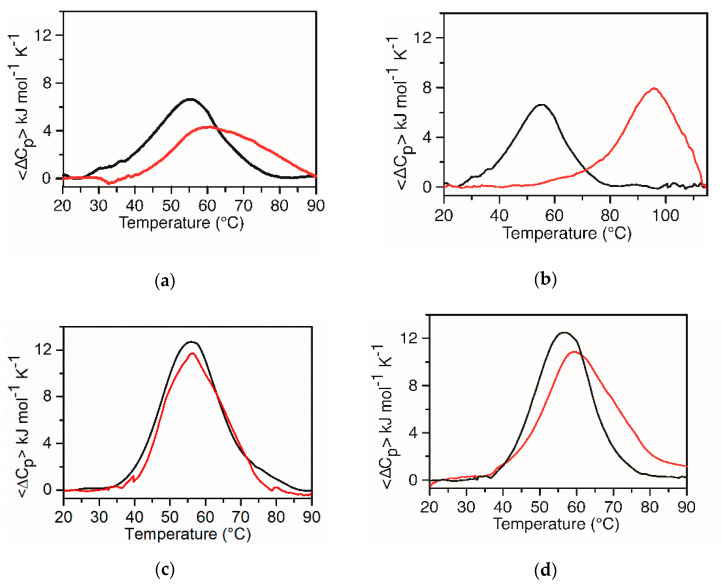
DSC profiles of *KRAS* 22RT (**a**) and *KRAS* 32R (**c**) in the absence (black line) and presence (red line) of 1 equivalent of TMPyP4. DSC profiles of *KRAS* 22RT (**b**) and *KRAS* 32R (**d**) in the absence (black line) and presence (red line) of 1 equivalent of BRACO-19. The buffer solution was 20 mM KH_2_PO_4_/K_2_HPO_4_ with 60 mM KCl and 0.1 mM EDTA at pH 7.0.

Focusing the attention on BRACO-19, whose interaction with *KRAS* G4s has not been thoroughly studied so far, we performed calorimetric experiments by DSC on both G4s in the presence of one equivalent of the ligand. The DSC profiles of both *KRAS* 22RT and *KRAS* 32R show interesting features (Figure 6b,d). Indeed, *KRAS* 22RT in the presence of the ligand shows a main peak centered at a higher temperature and a barely perceptible, but evident, shoulder at a lower temperature, confirming the distribution of a bound G4 with different stoichiometries. As far as *KRAS* 32R is concerned, the DSC curve of *KRAS* 32R displays a main peak centered at ≈ 58 °C with a shoulder at ≈ 75 °C, suggesting the different stability of the two conformers bound to BRACO-19. In addition, the large difference between the two temperatures, compared to those obtained for G4 alone, suggests that one of the two conformers is more stabilized by the interaction with BRACO-19. A comparison with the CD melting curve in the presence of one equivalent of the ligand (Figure 5d, red line) shows that the DSC profile is richer in information. The CD melting profile appears monophasic, and it is not descriptive of the complex unfolding transition of *KRAS* 32R in the presence of BRACO-19. From now on, this should always be taken into consideration when studying *KRAS* 32R-targeting compounds. We also performed DSC experiments on both G4s in the presence of one equivalent of TMPyP4; the calorimetric curves are shown in Figure 6a,c. The DSC data confirms the CD results; TMPyP4 does not stabilize *KRAS* 32R, whereas a stabilization of 4 °C was found for *KRAS* 22RT in our solution conditions. The presence of the ligand produces also a broadening of the curve.

## 3. Discussion

In this paper, spectroscopic and calorimetric experiments were performed to study the stability of *KRAS* 22RT and *KRAS* 32R G4-forming sequences within the *KRAS* NHE sequence in the promoter region.

DSC is a well-known and powerful methodology for studying the stability of biomacromolecules and has been successfully applied to G4s [35,36,37,38]. In this study, DSC measurements were performed to obtain model-independent thermodynamic parameters relative to the unfolding processes of *KRAS* 22RT and *KRAS* 32R and to compare their relative stability. Calorimetric *T*_m_ and enthalpy changes were compared to those obtained by CD melting curves and obtained by the van ’t Hoff analysis, assuming a two-states unfolding process. The melting process of *KRAS* 22RT follows a two-states transition: the Δ_exp_*H°*/Δ_v.H._*H°* ratio is one, and Δ_exp_*H°* falls in the range of the awaited value, considering an average contribution of ∼50–80 kJ mol^−1^ for each G-tetrad, depending on the presence or none of loops [39].

On the other hand, the melting process for *KRAS* 32R is more complex. The curve was deconvoluted with a thermodynamic model based on the presence of two conformers in equilibrium with the unfolded state, according to the recent NMR studies [17]. The agreement between the experimental and calculated curves is satisfactory (Figure 4). The overall stability of *KRAS* 32R is greater than that of *KRAS* 22RT, as to be expected for the presence of ten additional nucleotides that can form additional interactions. However, the direct comparison cannot be made without analyzing the different stability of the two conformers of *KRAS* 32R. They show very close melting temperatures (56 °C and 57 °C); however, these values derived from the ratio between very different values of enthalpy and entropy changes (*T*_m_ = Δ*H*°/Δ*S*°). Both conformers were shown to have three G-tetrads and a parallel strand orientation but different topological arrangements of loops [17]. The conformer with a higher enthalpy change is stabilized by a greater number of interactions and, from NMR studies, can be univocally attributed to the structure with an additional triad formed by two guanines and one adenine at the 3′ end. The triad is stabilized by H-bonds and by π-π-stacking interactions with the adjacent tetrad of guanines. These additional interactions increase the enthalpy change that is largely compensated by a decrease in entropy change accompanying the optimization of the backbone structure. The other conformer, as suggested by the thermodynamic studies and according to the NMR structure, is more flexible and, consequently, less stable. In agreement with the NMR results reported in the literature [17], we found that, at 37 °C, both conformers are significantly populated with an excess of the more stable conformer (Figure S3).

Regarding the interactions with the investigated ligands, the stabilizing effect of TMPyP4 on *KRAS* 22RT is very low in the solution conditions used in this study (Δ*T*_m_ = 2 °C and 3 °C in the presence of one and two ligand equivalents, respectively) but in line with a previous work where we found a Δ*T*_m_ of 11 °C with eight equivalents of the ligand [23]. Surprisingly, TMPyP4 is unable to stabilize *KRAS* 32R, not even raising the ratio to five equivalents in the solution conditions here employed (Figure S5). The CD results are confirmed by DSC measurements. Conversely, Cogoi et al. found, by fluorescence resonance energy transfer (FRET) experiments, that TMPyP4 should stabilize *KRAS* 32R in the Tris/HCl buffer at pH 7.4 and 100 mM KCl [14]. Here, we used the exact same G-forming sequence but in phosphate buffer at pH 7.0 and 60 mM KCl, solution conditions very close to those utilized for the NMR studies. Furthermore, it should be pointed out that, in FRET melting experiments, the ligand molecule may interfere with fluorescent probes rather than with the DNA. In that case, an increase in melting temperature would reflect an interaction with the fluorophores, not with the target structure, generating false positives [40,41]. In the same paper, Cogoi et al. also suggested the presence of two conformations (parallel and antiparallel) of *KRAS* 32R with very different melting temperatures at around 55 °C and 72 °C, respectively. We found that the two conformers were both in parallel conformations (and with similar melting temperatures); indeed, no peak at 295 nm was highlighted in CD spectra recorded in the absence and presence of the ligands.

As far as BRACO-19 is concerned, we found that it increases the thermal stability of both G4s, as measured by the change in thermal melting temperature (Δ*T*_m_), but with a different stabilizing effect (Figure 5b,d). The DSC profile shows that the melting temperatures of both the conformers of *KRAS* 32R increase in the presence of BRACO-19, but the increase is greater for one of the two.

These thermodynamic studies shed light on the different stability of *KRAS* 22RT, *KRAS* 32R and its conformers, in terms of changes in Gibbs energy, entropy and enthalpy, and show that BRACO-19 stabilizes both the structures and bind both the conformers of *KRAS* 32R with the preference of one of them. Interestingly, BRACO-19 was found to stabilize G4 structures over double- and single-stranded DNA [42], whereas TMPyP4 is not selective for G4s and binds also the DNA duplex [18,43].

The different stability of the conformers could have some implications in biological processes—for example, in modulating the binding of the proteins to the promoter, as also suggested by Salgado et al. [17]. In addition, the less stable and more flexible conformer could be better resolved by specific helicases. Therefore, the next challenging studies should be to discover what is in vivo the predominant G4 structure among *KRAS* 22RT or one of the two conformers of *KRAS* 32R. Our study and previous ones seem to point towards *KRAS* 32R in terms of greater stability and conformational versatility.

## 4. Materials and Methods

### 4.1. Materials

The sequences d(AGGGCGGTGTGGGAAGAGGGAAGAGGGGGAGG) (*KRAS* 32R) and d(AGGGCGGTGTGGGAATAGGGAA) (*KRAS* 22RT) were purchased from Biomers.net GmbH (Ulm, Germany). TMPyP4 ((5,10,15,20-tetrakis(*N*-methyl-4-pyridyl)porphyrin)) and BRACO-19 (*N*,*N*’-(9-[(4-(dimethylamino)phenyl)amino]acridine-3,6-diyl)bis(3-(pyrrolidin-1-yl)propanamide)), as well as all common chemicals, reagents and solvents, were purchased from Sigma Aldrich (Merck KGaA, Darmstadt, Germany) unless otherwise stated.

### 4.2. DNA Samples Preparation

Oligonucleotide samples were prepared by dissolving the lyophilized DNAs in potassium phosphate buffer (60 mM KCl, 20 mM KH_2_PO_4_/K_2_HPO_4_ and 0.1 mM EDTA, pH 7.0). The solutions were heated at 90 °C for 5 min and slowly cooled to room temperature. The concentration of the oligonucleotides was evaluated by UV measurements at 260 nm at a temperature of 90 °C, using molar extinction coefficient values calculated by the nearest-neighbor model [44].

### 4.3. Circular Dichroism

CD spectra were recorded on a Jasco J-815 spectropolarimeter (JASCO Inc., Tokyo, Japan) equipped with a PTC-423S/15 Peltier temperature controller. All the spectra were recorded at 20 °C in the 220–360 nm wavelength range and averaged over three scans. The scan rate was 100 nm min^−1^, with a 4 s response and 1 nm bandwidth. Buffer baseline was subtracted from each spectrum. Sample concentration was 2 µM for all DNA samples. CD melting experiments were carried out in the 20–100 °C range at 0.5 °C min^−1^ and 1 °C min^−1^ heating rates following changes of the CD signal at the wavelength of maximum intensity (264 nm for *KRAS* 22RT and 263 nm for *KRAS* 32R). Three-dimensional melting curves for G4s were also obtained by recording whole CD spectra every two degrees with the same parameters previously described. The melting temperatures (*T*_m_) were determined from the curve fit using Origin 7.0 software (OriginLab Corp., Northampton, MA, USA). Moreover, CD spectra and melting experiments were recorded both in the absence and presence of each ligand. G4/ligand mixtures were obtained by adding 1 or 2 mol equiv. (2 or 4 μM) of BRACO-19 and TMPyP4 to the folded G4 structures. Δ*T*_m_ values were determined as the difference in the melting temperature of the G4 structures with and without ligands. All experiments were performed in duplicate, and the reported values were the average of two measurements.

### 4.4. Differential Scanning Calorimetry

DSC measurements were carried out on a nano-DSC (TA Instruments, New Castle, DE, USA) using a 200–400 μM G4 sample in a 60 mM KCl, 20 mM KH_2_PO_4_/K_2_HPO_4_ and 0.1 mM EDTA buffer at pH 7.0. Three heating/cooling cycles were recorded in the 10–100 °C range, 0.5 °C/min and 1 °C/min scan rate, using a 600 s equilibration time prior to each heating and 300 s before each cooling. The same method was used for buffer versus buffer scans to obtain the baseline, which was subtracted from sample versus buffer scan to obtain the thermodynamic parameters. The corrected thermograms were then normalized per mole of DNA to obtain the corresponding molar heat capacity curves. Model-free enthalpy, Δ_exp_*H°*, for the overall unfolding of the DNA structure was estimated by integrating the area under the heat capacity versus temperature curves and representing the average of at least three different heating experiments. *T*_m_ values corresponded to the maximum of each thermogram peak. DSC curves provided the van ‘t Hoff enthalpy (Δ_v.H._*H°*), calculated assuming a simple two-states transition. The same experiment was carried out by adding TMPyP4 or BRACO-19 (1 or 2 mol equiv.) to the folded G4 structures. To exclude the possible decomposition of BRACO-19 and TMPyP4 upon increasing the temperature, we performed UV measurements in the spectral region from 220 nm to 700 nm at 10 °C and 100 °C. The spectra were quite similar, showing the stability of the ligands also at high temperatures (Figure S6).

#### 4.4.1. Thermodynamic Model

To analyze the *KRAS* 32R DSC profile, we developed a model to describe a system where the DNA exist in a mixture of two independently folded states, Q1 and Q2, that unfold to the state U with equilibrium constants *K*_1_ and *K*_2_. Notably, the equilibrium constant for the interconversion of the two folded states is not another independent parameter (it is given by the ratio *K*_1_/*K*_2_). The canonical partition function for such a system can be written as a function of the species concentration as follows:

(1) $$Q\left(T\right)\;=\;\left[Q_{1}\right]\;+\;\left[Q_{2}\right]\;+\;\left[U\right]\;=\;\frac{\left[U\right]}{K_{1}}\;+\;\frac{\left[U\right]}{K_{2}}\;+\;\left[U\right]$$

It is convenient for such a system to choose as the reference state the common unfolded state U; in this case, the relative partition function was:

(2) $$Q_{U}\left(T\right)\;=\;\frac{K_{1}\;+\;K_{2}\;+\;K_{1}K_{2}}{K_{1}K_{2}}$$

The molar fractions of the species were given by:

(3) $$f_{1}\;=\;\frac{K_{1}}{Q_{U}}$$

(4) $$f_{2}\;=\;\frac{K_{2}}{Q_{U}}$$

(5) $$f_{U}\;=\;\frac{1}{Q_{U}}$$

The excess enthalpy of the system relative to the enthalpy of the unfolded states was obtained by the relative partition function by means of the well-known relation of the statistical thermodynamics:

(6) $$<\Delta H\left(T\right){>}_{U}\;=\;RT^{2}\left(\frac{\partial lnQ_{U}}{\partial T}\right)$$

By performing the derivative in Equation (6) at a fixed total DNA concentration, it was possible to obtain the following analytical expression for excess enthalpy:

(7) $$<\Delta H\left(T\right){>}_{U}\;=\;-\left(\Delta H_{1}^{0}f_{1}+\Delta H_{2}^{0}f_{2}\right)$$

where $\Delta H_{i}^{0}$ and $f_{i}$ were the unfolding enthalpy and the molar fraction of the i-folded state. Finally, the excess heat capacity, with respect to the unfolded or folded states (assuming negligible differences in the heat capacity for the three states), was obtained by deriving Equation (7), numerically or analytically, with respect to the temperature:

(8) $$\langle \Delta C_{p}\left(T\right)\rangle \;=\;\frac{d}{dT}\langle \Delta H\left(T\right)\rangle$$

The experimental DSC profiles were fitted by means of Equation (8) to give the enthalpy changes per mole of the species (Q1 or Q2) and the *T_m_* reported in Table 2.

## Figures and Tables

**Table 1:** Thermodynamic parameters calculated by the van ’t Hoff analysis of the circular dichroism (CD) melting curves.

	*T*_m_ (°C) ^1^	Δ_v.H._*H*° (kJ mol^−1^) ^1^
*KRAS* 22RT	52.0	200
*KRAS* 32R	59.2	220

^1^ The error on the melting temperature (*T*_m_) is ±0.5 °C and the error on Δ_v.H._*H*° is ±5%.

**Table 2:** Thermodynamic parameters obtained by the differential scanning calorimetry (DSC) profiles.

	*T*_m_ (°C)	Δ_exp_*H*° (kJ mol^−1^)	Δ_calc_*H*° (kJ mol^−1^)	Δ*S°* (kJ mol^−1^ K^−1^)	Δ*G*°_(310 K)_ (kJ mol^−1^)
*KRAS* 22RT	54.4	208	207	0.636	10.8
*KRAS* 32R	56.2 57.2	270	265 * 140 *	0.805 0.424	15.5 8.6

The error on the *T*_m_ is ±0.5 °C, on Δ*H*° and Δ*S*° is ±5%, and on Δ*G*°_(310 K)_ is ±10%. * These enthalpy changes are expressed per mole of Q1 or Q2.

## Data Availability

The data presented in this study are available on request from the corresponding author.

## Supplementary Materials

The supplementary materials can be found at https://www.mdpi.com/1422-0067/22/1/448/s1.

## Abbreviations

CD	circular dichroism
DSC	differential scanning calorimetry
FRET	fluorescence resonance energy transfer
G4	G-quadruplex
*KRAS*	Kirsten ras
