# Supplementary material for: KRAS Promoter G-Quadruplexes from Sequences of Different Length: A Physicochemical Study
Source: Int J Mol Sci. 2021 Jan 5;22(1):448. doi: 10.3390/ijms22010448 (PMC7795837; doi:10.3390/ijms22010448)
Supplement: Supplementary file 1 [file ijms-22-00448-s001.pdf]

Supplementary Material

KRAS promoter G-quadruplexes from sequences of different length: a physicochemical study

Federica D'Aria [1], Bruno Pagano [1], Luigi Petraccone [2] and Concetta Giancola [1,*]

[1] Department of Pharmacy, University of Naples Federico II, Via D. Montesano 49, 80131 Naples, Italy; federica.daria@unina.it (F.D.); bruno.pagano@unina.it (B.P.);
[2] Department of Chemical Sciences, University of Naples Federico II, Via Cintia 4, 80126 Naples, Italy; luigi.petraccone@unina.it
* Correspondence: giancola@unina.it

**Table of contents**	**Page**
Figure S1	S2
Figure S2	S2
Figure S3	S3
Figure S4	S3
Figure S5	S4
Figure S6	S4

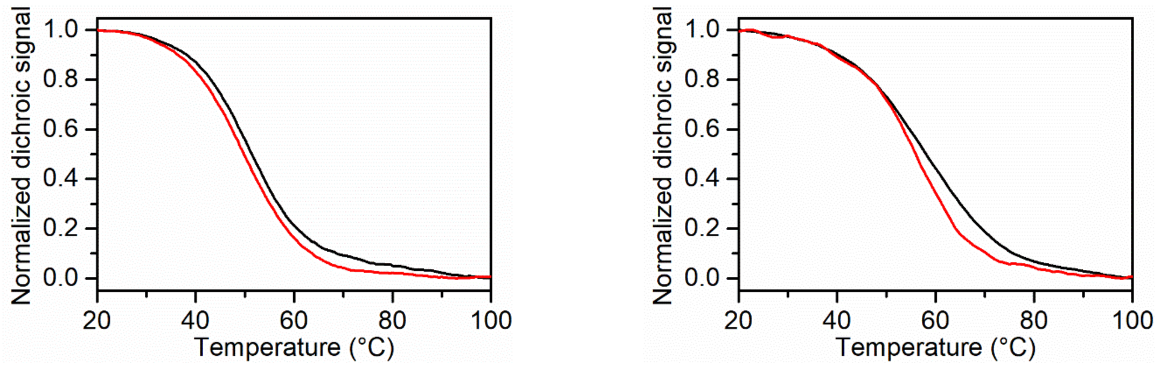

(a) (b)

Figure S1. CD melting (black line) and annealing (red line) profiles of (a) *KRAS* 22RT and (b) *KRAS* 32R at 1 °C min^{-1}.

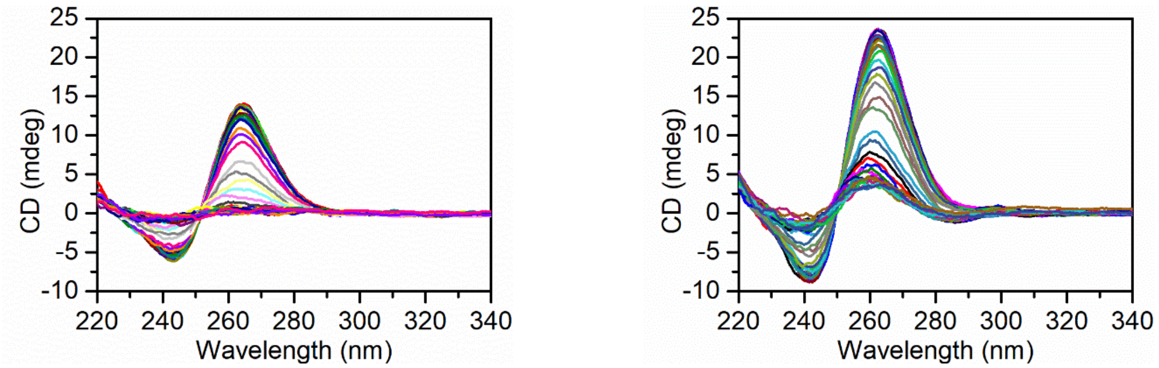

(a) (b)

Figure S2. CD spectra of (a) *KRAS* 22RT and (b) *KRAS* 32R collected as function of temperature.

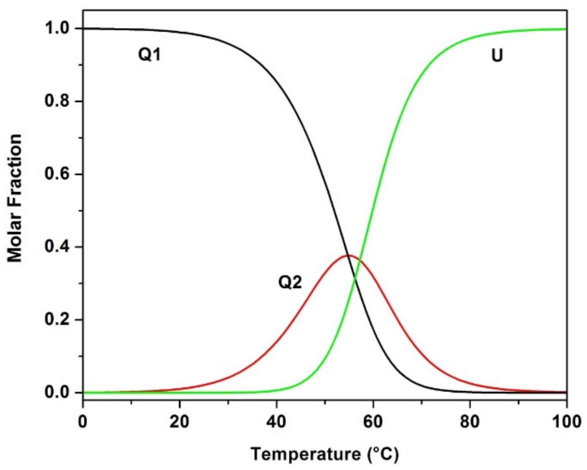

Figure S3. Molar fractions of Q1 (black), Q2 (red) and U (green) as function of temperature.

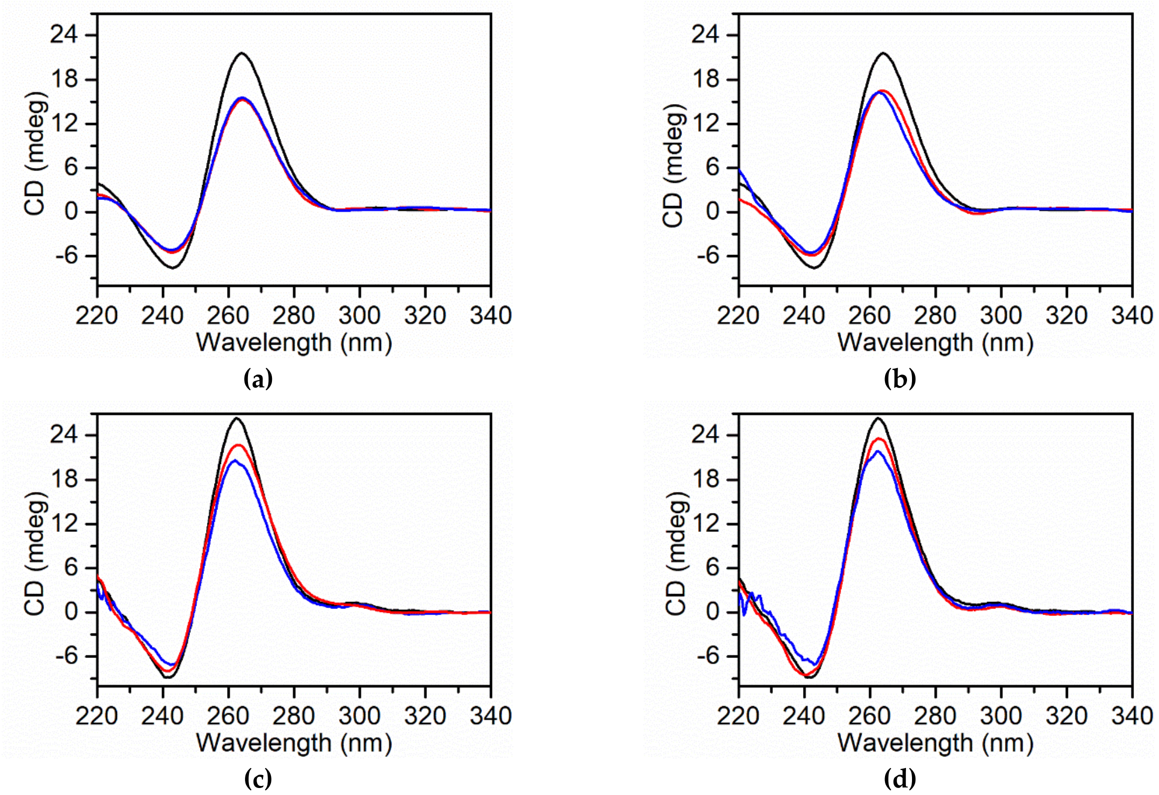

Figure S4. CD spectra of (a) *KRAS* 22RT and (c) *KRAS* 32R in the absence (black line) and in the presence (red line) of 1 (red line) and 2 equivalents (blue line) of TMPyP4. CD spectra of (b) *KRAS* 22RT and (d) *KRAS* 32R in the absence (black line) and in the presence of 1 (red line) and 2 equivalents (blue line) of BRACO-19.

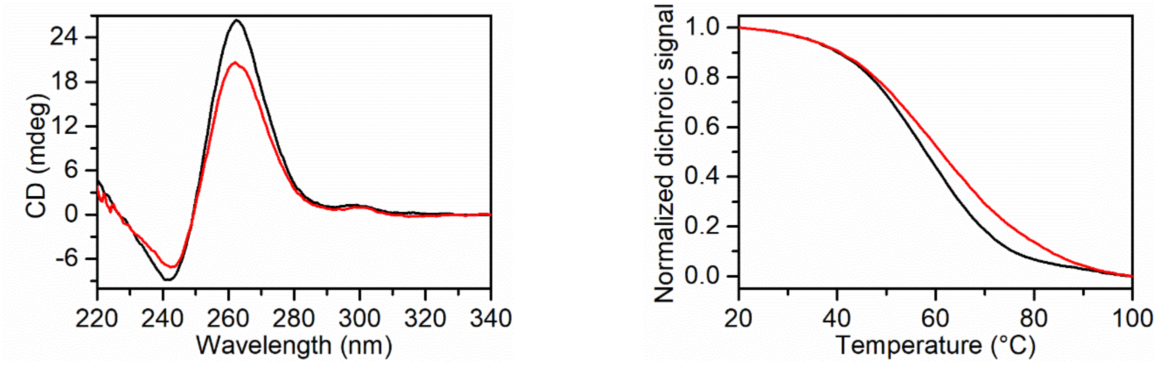

(a) **(b)**

Figure S5. CD spectra (a) and CD melting curves (b) of *KRAS* 32RT in the absence (black line) and in the presence (red line) of 5 equivalents of TMPyP4.

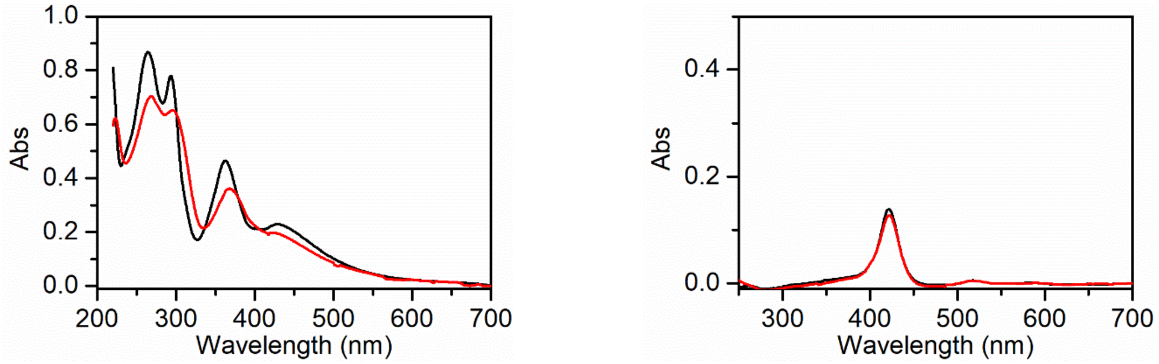

(a) **(b)**

Figure S6. UV/visible spectra of BRACO-19 and TMPyP4 at 10°C (black line) and 100 °C (red line).
